# Spatial patterns of natural hazards mortality in the United States

**DOI:** 10.1186/1476-072X-7-64

**Published:** 2008-12-17

**Authors:** Kevin A Borden, Susan L Cutter

**Affiliations:** 1 Hazards and Vulnerability Research Institute, Department of Geography, University of South Carolina, Columbia, SC 29208, USA

## Abstract

**Background:**

Studies on natural hazard mortality are most often hazard-specific (e.g. floods, earthquakes, heat), event specific (e.g. Hurricane Katrina), or lack adequate temporal or geographic coverage. This makes it difficult to assess mortality from natural hazards in any systematic way. This paper examines the spatial patterns of natural hazard mortality at the county-level for the U.S. from 1970–2004 using a combination of geographical and epidemiological methods.

**Results:**

Chronic everyday hazards such as severe weather (summer and winter) and heat account for the majority of natural hazard fatalities. The regions most prone to deaths from natural hazards are the South and intermountain west, but sub-regional county-level mortality patterns show more variability. There is a distinct urban/rural component to the county patterns as well as a coastal trend. Significant clusters of high mortality are in the lower Mississippi Valley, upper Great Plains, and Mountain West, with additional areas in west Texas, and the panhandle of Florida, Significant clusters of low mortality are in the Midwest and urbanized Northeast.

**Conclusion:**

There is no consistent source of hazard mortality data, yet improvements in existing databases can produce quality data that can be incorporated into spatial epidemiological studies as demonstrated in this paper. It is important to view natural hazard mortality through a geographic lens so as to better inform the public living in such hazard prone areas, but more importantly to inform local emergency practitioners who must plan for and respond to disasters in their community.

## Background

Outcomes of natural hazard events can be grouped into two general categories; economic losses (including property, agricultural, direct, and indirect losses) and casualties (injuries and fatalities). Despite these two potential impacts on populations, contemporary hazards research in the United States focuses more on economic losses and loss reduction rather than examining casualties. This bias reflects the downward trend in casualties and the dramatic increases in hazard losses over time in the United States and other more developed countries [1,2]. Despite the downward trend in human casualties, developed countries are still susceptible to significant losses of life from natural hazard events as shown by Hurricane Katrina, the 2003 European heat wave, and the 1995 Chicago heat wave.

Previous hazard mortality studies often lack a breadth of hazard types and utilize limited geographic scales. Notable exceptions include a global risk analysis that includes various hazard types [3], and a U.S. based historical analysis of hazard mortality [4]. However, researchers often examine deaths for only one particular type of hazard such as floods [5,6], earthquakes [7,8], tornadoes [9], or heat [10]. Although detailed examination of hazard related deaths for one hazard type is important, this fragmented and unitary approach leaves many unanswered questions about the geography of deaths from natural hazards as a whole. It also limits comparability between hazard event types. Certain hazard types (e.g., heat, floods) often are described as the number one cause of hazard related death without an appropriate multi-hazard study to substantiate such claims. Indeed, claims of examining "the deadliest hazard" often are used as justification for studying the mortality associated with a particular hazard type.

This paper examines the spatial patterns associated with hazard mortality at a sub-state level for the United States using a combination of geographical and epidemiological methods, and a sub-county georeferenced hazards events and losses database, SHELDUS. Two specific research questions are examined: 1) Which natural hazard contributes most to hazard-induced mortality, and 2) What is the spatial patterning of natural hazard mortality in the United States?

### Studying death geographically

Studying any type of mortality is inherently geographical. Since the initial work of John Snow [11], countless mortality atlases have been produced such as those for, cancer [12], toxic hazards exposure [13], and all causes [14,15]. Mortality mapping permits the exploration of spatial patterns [16,17]; the development of more robust mortality mapping approaches [18,19]; testing for statistically significant spatial clusters of mortality [20,21]; and temporal analysis [22,23].

Research that examines the spatial aspects of mortality has grown significantly over time, forming a niche in spatial epidemiology, which merges spatial analysis techniques from geography with mortality studies from public health/epidemiology [18,24,25]. Within spatial epidemiology, considerable research effort has focused on the computation of robust measures of mortality [26,27], different clustering techniques to analyze spatial patterns [21,28], and the creation of a reliable map of disease or mortality that is free of spurious statistical variation [29].

### Natural hazard mortality

Despite the advancement of health geographics, the application of spatial epidemiological methods has not been applied systematically to deaths from natural hazards in the United States. Perhaps the biggest hindrance to conducting such broad spatial-analytical research on the geography of hazard deaths has been the lack of quality data. In order to explore hazard related deaths in a meaningful way, researchers need a large data repository that stores information on a variety of hazard types at a resolution fine enough to detect spatial patterns. A comprehensive, centralized, and reliable accounting of georeferenced natural hazard deaths has thus far been unavailable. Also the rarity of hazard deaths, especially in developed countries introduces the methodological issue of the "small number" problem resulting from calculating mortality rates for a rare cause of death in small areas.

Despite these limitations, there is an emerging literature on natural hazard mortality. For example, a number of studies have been conducted on the patterns of death in specific disaster events such as Hurricane Andrew [30,31], the Northridge Earthquake [32,33], and the Chicago heat wave [10]. These types of studies are useful for determining specific causal mechanisms between hazards and death, but out of necessity, they are highly localized, and event specific. Other research has focused on more general causes and circumstances of hazard mortality from specific hazard types such as floods [34], or heat [35], and generalized effects of climate on mortality [36,37]. Although informative and useful, the geography of hazard mortality is often an ancillary piece of the research, not the primary focus.

The spatial patterning of hazard mortality is less understood and studied. For example, Kalkstein and Davis [38] examined the effect of temperature on mortality using various cities throughout the United States as sample points, thereby providing a comparative regional analysis of urban areas. Two different studies examined tornado and flood deaths in the United States using spatially gridded data. A 40 km cell size was chosen to analyze flood deaths to approximate normal county size [5], yet a larger cell size was used for tornado deaths (60 km) without justification [9]. Although an interesting approach, questions remain on the reasoning behind the choice of a particular pixel size, and the lack of size consistency for studying deaths from different hazards. Finally, Thacker et al. [4] was one of the first studies to examine multi-hazard mortality analysis using the CDC's *Compressed Mortality File*. In terms of encompassing a broad range of natural hazard types, their work most closely resembles the scope of this paper. However, Thacker et al. [4] cover a shorter time period (1979–2004) and fail to provide a strong spatial component to their research despite having county-level data. They offer a tabular analysis of mortality rates for various regions in the United States, but fail to provide a systematic spatial analysis.

A review of the literature shows that some studies contain a spatial-analytic component but not a range of hazard types, while other studies examine multiple hazards but use aspatial techniques. A natural hazard mortality study that combines spatial analysis at a fine resolution for a wide variety of natural hazards is missing. This paper improves the spatial resolution and analytic techniques of previous studies and includes a broader range of natural hazards in the analysis, thus providing a more complete picture of the geography of natural hazards mortality in the U.S.

## Methods

### Data

The mortality data for this paper were culled from the Spatial Hazard Event and Loss Database for the United States (SHELDUS)(available at ). This database provides hazard loss information (economic losses and casualties) from 1960 – 2005 for eighteen different hazard types at county level resolution [39] for all 50 states. To maintain consistency in the county level enumeration units and the quality of the mortality data, three adjustments were made. First, Alaska and Hawaii were excluded from the analysis. Second, to maintain consistent geographic units through time any changes in county boundaries were attributed to the original county for the entire time-period (this includes counties that were split or merged). Finally, all independent cities in Virginia, Maryland, and Missouri were absorbed into their respective counties. After these modifications, 3,070 county level enumeration units were used in this study.

Inconsistencies in the SHELDUS database were first addressed before any mortality measures were constructed. For the implementation of spatial epidemiological methods, two problems embedded in the design of SHELDUS warrant a brief discussion. These include event thresholds and geographic attribution of deaths.

These event thresholds were due to the reliance in SHELDUS on its primary data source, NCDC's *Storm Data*, which reported events on a categorical damage scale (Table 1) [39,40]. Prior to 1995, only events that generated at least $50,000 (Category 5) in economic damage were included in the SHELDUS database. SHELDUS used the lower bound of each damage category when reporting losses to maintain the most conservative estimate of losses possible. After 1995, however, NCDC increased the precision of loss information for hazard events, reporting losses as exact dollar amounts rather than logarithmic categories. Thus, SHELDUS contained two time-periods with markedly different standards regarding which events were included in the database. For example, prior to 1995, mortality from events that failed to reach the monetary threshold (such as lightning) would be excluded. To adjust for this problem, any event in *Storm Data *that caused a death and less than $50,000 in economic damages was added to the SHELDUS database for the period 1970–1995. Correcting these problems for 1960–1969 is currently underway by the SHELDUS developers and was unavailable for use in this paper.

**Table 1:** NCDC damage categories

Category	Damage Range
1	< $50
2	$50 – $500
3	$500 – $5,000
4	$5,000 – $50,000
5	$50,000 – $500,000
6	$500,000 – $5,000,000
7	$5,000,000 – $50,000,000
8	$50,000,000 – $500,000,000
9	$500,000,000 – $5,000,000,000

The second issue with the original SHELDUS data is the geographic attribution of deaths. When events affected multiple counties, and there was no information on the specific county where the fatality or the monetary losses occurred, all losses and casualties were evenly distributed across the affected counties, leading to fractional deaths and injuries [39]. After 1995, however, *Storm Data *became more geographically precise in defining the locations of events, and the attribution of those losses and deaths to specific counties. Accordingly, SHELDUS was more deliberate in attributing deaths to their proper geographic location. This inconsistency necessitated a quality control analysis on SHELDUS data prior to 1995. From 1970 to 1995, every event in SHELDUS with a death was verified against *Storm Data *for geographic accuracy. In instances where the county of death was specified in *Storm Data*, SHELDUS was changed to reflect that information.

### Mapping

The simplest way to characterize mortality in the form of a rate is to map crude rates by dividing the number of deaths by the population at risk (usually the mid-year population) [41,42]. Although crude rates can indicate where the magnitude of deaths is large [43], a major drawback is that they do not account for differences in the population structure of different areas [43]. To account for varying age structures between counties, we employed indirect age standardization to our data and calculated standardized mortality ratios (SMRs) for each county. Indirect standardization was necessary for this analysis because SHELDUS data lacks the age at death for hazard fatalities. Although there is debate in the epidemiologic literature as to the utility of SMRs [44], they are a widely used and accepted measure of mortality in spatial epidemiology research [18,27,44].

Often referred to as the small number problem, spurious variation in rates can result from small denominator data (i.e. population) [45,46]. Counties with small populations demonstrate extremely high mortality rates when, in fact, there are few actual recorded deaths. Furthermore, greater than expected fluctuation in mortality rates occurs with the addition of only one or two extra cases in low population counties. To adjust for spurious variation without compromising spatial resolution, mortality rates were transformed using an empirical-bayes operation to remove artificial extreme values, yet maintain the structure of broad spatial trends. This technique is commonly used with small-area rate data and is encouraged over regular SMRs [18].

To analyze the observed patterns spatially, we employed a local cluster analysis on the map of hazard mortality. The local Moran's I statistic [47] provided in the GeoDa 0.9.5-i5 software package [48] was used to reveal contiguous areas of elevated mortality. Such local statistics are useful to analyze the spatial variation of clusters that are not apparent in global measures.

### Data classification changes

To better interpret SHELDUS deaths across hazard types, the 18 original hazards were generalized into 11 distinct categories (Table 2). For the purposes of analyzing deaths across hazard categories, each event, regardless of how many hazards were involved was assigned into one and only one category. For example, an event with multiple causes (e.g. hail, wind, lightning, and rain) was categorized as Severe Weather. Even though lightning is associated with severe weather, it has its own category due to the number of fatality inducing events reported with lightning as the only hazard.

**Table 2:** Generalized SHELDUS hazard types

***SHELDUS Category***	***Generalized Category***
Coastal	Coastal (e.g. storm surge, rip currents, coastal erosion)
Flooding	Flooding (e.g. flash, riverene)
Earthquake	Geophysical
Tsunami/Seiche	
Volcano	
Drought	Heat/Drought
Heat	
Hurricane/Tropical Storm	Hurricane/Tropical Storm
Lightning	Lightning
Avalanche	Mass Movement
Landslide	
Fog	Severe Weather
Hail	
Severe Storm/Thunderstorm	
Wind	
Tornado	Tornado
Wildfire	Wildfire
Winter Weather	Winter Weather

As a result of adding deaths from events that generated less than $50,000 in damages, the total number of fatalities from 1970 – 2004 increased by 31% (Table 3). As expected, lightning was the most affected category based on the new corrections, increasing its number of fatalities by 77%. This clearly demonstrates databases that record hazard losses based solely on economic damages may not be the most appropriate ones to use when studying hazard mortality without first correcting for errors of omission.

**Table 3:** Effect of fatality corrections in SHELDUS

***Event Type***	***SHELDUS***	***Mortality Database***	***Total***	***Increase***
Lightning	517	1,744	2,261	77%
Winter Weather	2,306	1,306	3,612	36%
Severe Weather	2,402	1,360	3,762	36%
Coastal	357	99	456	22%
Flooding	2,188	600	2,788	22%
Heat/Drought	3,227	679	3,906	17%
Tornado	2,006	308	2,314	13%
Mass Movement	154	16	170	9%
Hurricane/Trop. St.	279	25	304	8%
Geophysical	302	0	302	0%
Wildfire	84	0	84	0%
**Totals**	**13,821**	**6,137**	**19,958**	**31%**

## Results

### Deadliest hazard types

**Figure 1:**
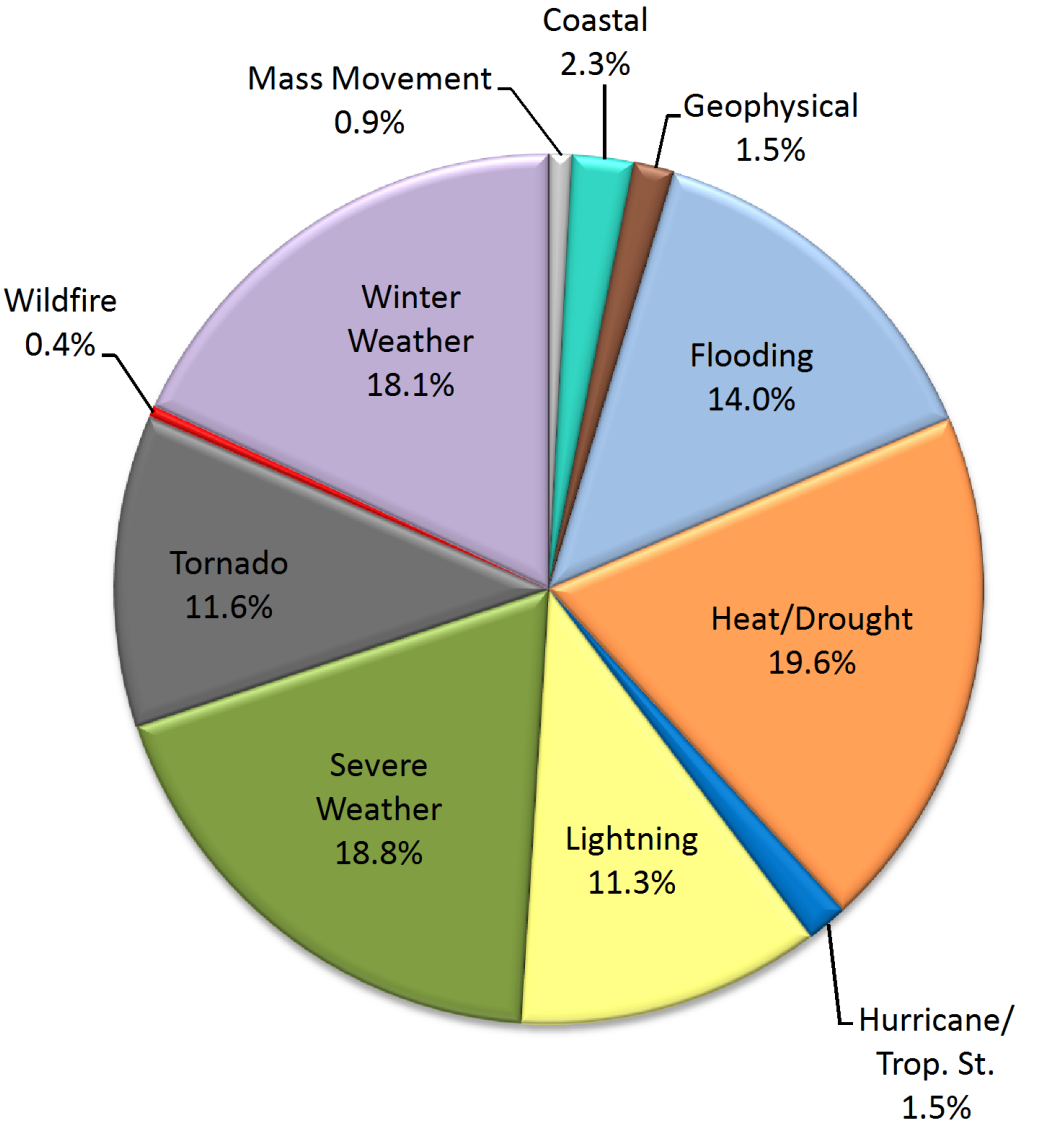
Natural hazard deaths by event type.

Figure 1 shows the distribution of deaths for 11 hazard categories as a percent of total hazard deaths from 1970 – 2004. Heat/drought ranks highest among these hazard categories causing 19.6% of total deaths, closely followed by severe summer weather (18.8%) and winter weather (18.1%). Geophysical events (such as earthquakes), wildfires, and hurricanes are responsible for less than 5% of total hazard deaths combined. What is noteworthy here is that over time, highly destructive, highly publicized, often catastrophic singular events such as hurricanes and earthquakes are responsible for relatively few deaths when compared to the more frequent, less catastrophic events such as heat waves, and severe weather (summer or winter).

### Spatial distribution

Using the corrected SHELDUS data, natural hazard mortality was mapped to visually illustrate its geographic distribution. We first aggregated the data to a regional scale using the geographic divisions of the Federal Emergency Management Agency (FEMA), and then we produced a county-level map. Comparing county-level mortality maps to those at a higher level of spatial aggregation serves two analytical purposes. First, similar spatial trends between the county and the aggregated regional map (which provide stable mortality estimates) increase the confidence that stability was achieved in the transformed county-level maps. Second, similar patterns at different spatial scales support the notion that the observed county-level patterns are not a function of scale-dependent processes, thereby increasing the confidence that this is an accurate representation of hazard-induced mortality. These county-level maps not only provide a stable mortality map for reference purposes, but also present hazard mortality estimates at enumeration units that are relevant to local emergency managers and public health officials.

Mortality data were indirectly standardized to the year 2000 national hazard mortality rate using standardized mortality ratios. At the county level, adjusted SMRs were calculated using the empirical bayes procedure provided in GeoDa 0.9.5-i5 [48] and log-transformed to achieve a normal distribution. The data were mapped using standard deviations from the mean.

**Figure 2:**
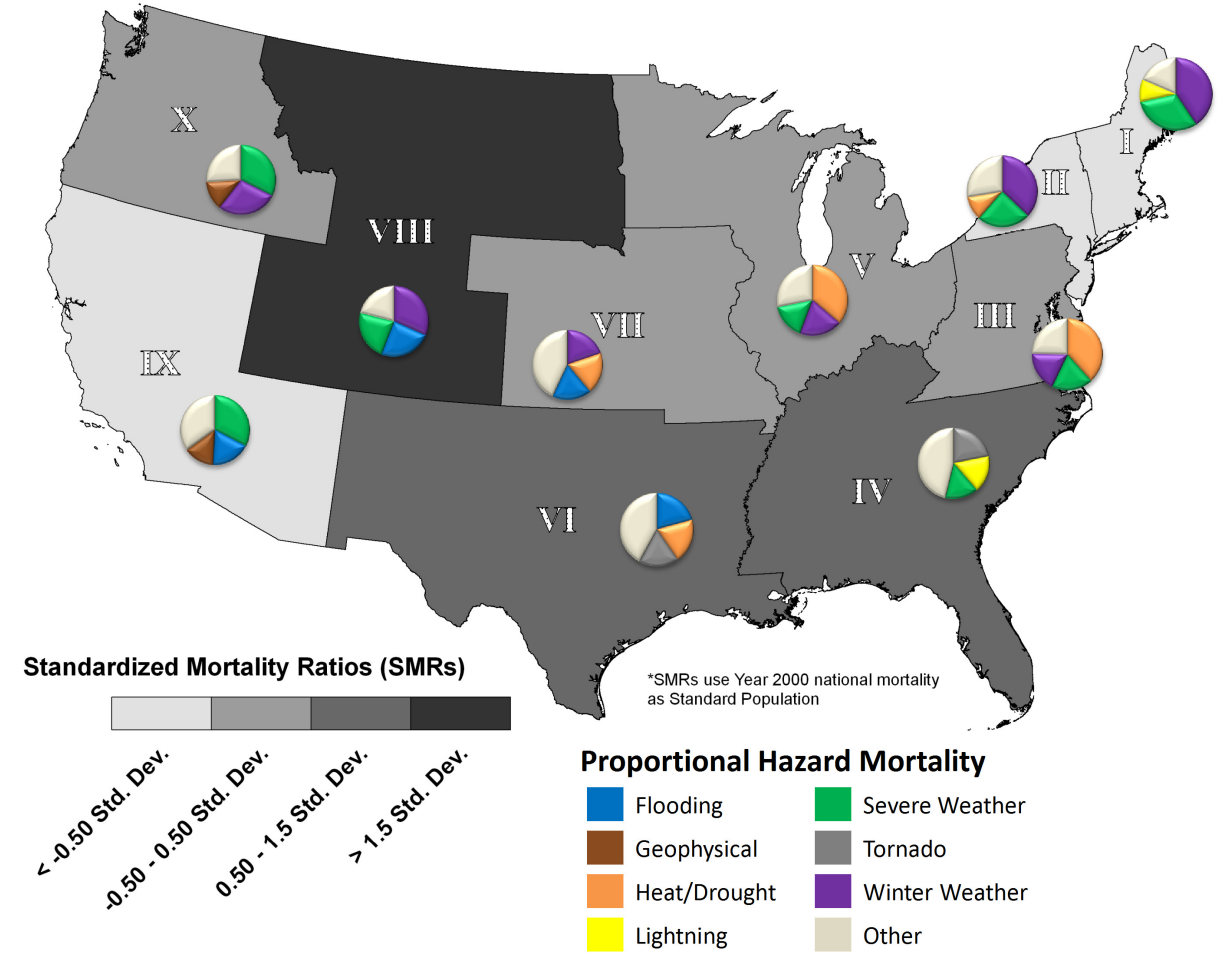
**Hazard induced mortality by FEMA region 1970 – 2004**. *SMRs use Year 2000 as Standard Population.

Hazard mortality is most prominent in the South (FEMA regions IV and VI) (Figure 2). While FEMA region VIII appears to have the highest risk level based on SMRs, this finding must be interpreted with caution, and is likely a function of the small population size within the region. Although the SMRs were stabilized with the empirical bayes procedure, hazard deaths are so rare that the small number problem cannot be totally removed. These regional patterns are due to the occurrence of various severe weather hazards and tornadoes (region IV), winter weather (region VIII), and floods and tornadoes (region VI) as the primary causal mortality agents. Pie charts located in each region show the proportion of the top three causes of hazard deaths. The fourth section in each pie chart, labeled "other", includes any of the generalized event types listed in Table 2. The utility of this classification approach is to visualize the relative significance of the top three causes of death in each FEMA Region. A FEMA Region with a very small "other" category as in Region 1 (Figure 2) suggests that the top three causes of death are quite important to the overall impact of hazard deaths. However, a large "other" category shows places such as Region 4 (Figure 2), where deaths are more evenly distributed by hazard type. Although potentially useful at the national emergency management level for assessing areas of higher mortality, the regions are so large that they do not show spatial variability in hazard mortality. While, the South shows elevated mortality, the higher mortality rates may be clustered in a few high- risk areas, not uniformly distributed throughout the region as the map suggests.

**Figure 3:**
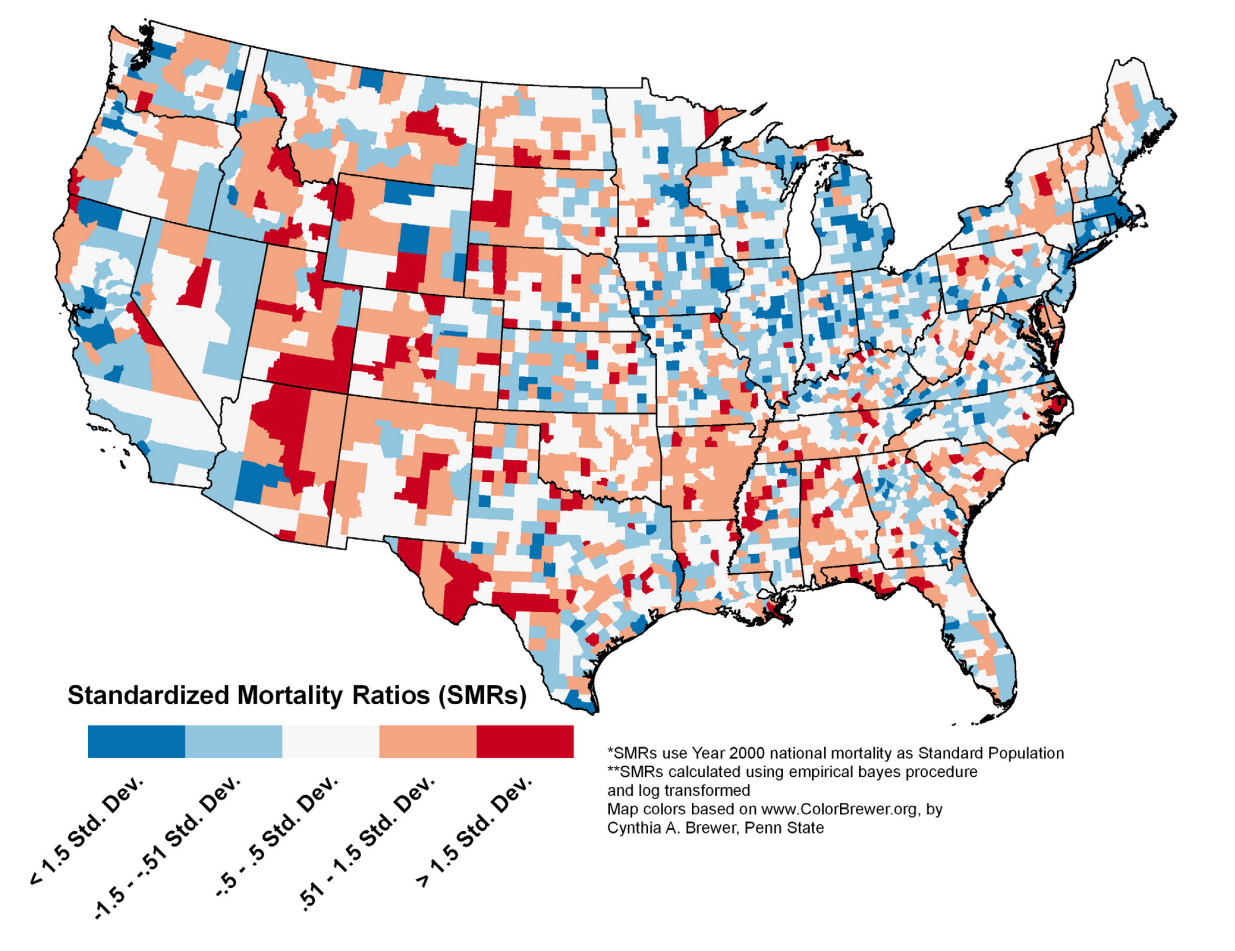
**County-level hazard induced mortality 1970 – 2004**. *SMRs use Year 2000 as Standard Population. **SMRs calculated using empirical bayes procedure and log transformed. Map colors based on , by Cynthia A. Brewer, Penn State.

County level hazard-induced mortality for the contiguous United States shows more spatial variability than the regional map (Figure 3). For instance, the highest values in the South are along the Atlantic and Gulf Coasts, especially in the Florida panhandle, and along the Carolinas' coast. Elevated mortality in southwestern Texas and throughout Arkansas contributes to higher mortality for FEMA region VI.

The initial visual analysis is suggestive of a regional patterning of mortality from natural hazards. However, the identification of clusters of elevated mortality should be achieved through local spatial statistics rather than simple visual interpretation because size, shape, and potential for spurious rate variation of polygons can create the illusion of clusters that are not statistically significant [49].

### Cluster analysis

To test for the presence of spatial clusters of hazard mortality, we employed spatial autocorrelation on the county level SMRs using both global and local indicators using the GeoDa. 0.9.5-i5 software package [48]. A global Moran's I test was performed to assess whether the pattern of SMRs had an average tendency to cluster in space [50]. Neighbors were designated based on first order queen contiguity [48]. The likelihood of positive spatial autocorrelation in the dataset was confirmed with a global Moran's I coefficient of .30 (p < .001).

**Figure 4:**
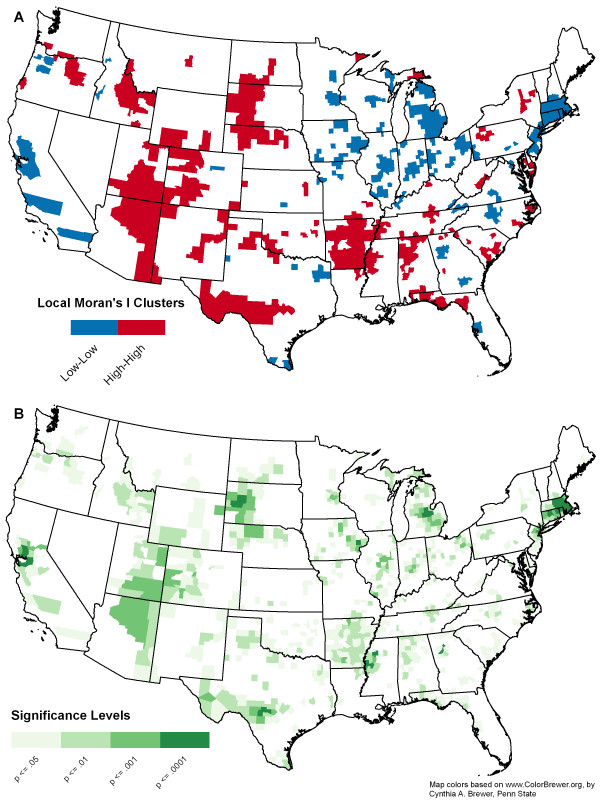
**County-level SMR clusters (A) and significance levels (B) from hazard induced mortality 1970 – 2004**. Map colors based on , by Cynthia A. Brewer, Penn State.

With a tendency for similar SMR values to cluster established, a local indicator of spatial association was used to identify the location of clusters. Local pockets of positive spatial autocorrelation (areas where similar values are clustered in space) for county level SMR data appear throughout the continental United States) (Figure 4a). Areas of high SMR values occur through the northern Plains, mountain west, and South, particularly the Florida panhandle, the Carolinas, lower Mississippi River, and Rocky Mountain west. Other noticeable trends include the tendency for large urban centers to demonstrate clusters of low SMR values (e.g. Atlanta, San Francisco, and New York). Low SMRs in urban areas do not mean that there is less overall risk, but instead less risk of dying on an individual basis since there are more people. Clusters of low SMR values generally occur in the Midwest and Northeast coastal corridor. All local clusters shown are statistically significant at 95% confidence. However, a confidence map shows variation in significance values to identify clusters that exceed 95% confidence (Figure 4b).

The local Moran's I and significance maps confirm our visual analysis of the geographic distribution of mortality from natural hazards. Those county level natural hazard mortality patterns most statistically relevant in terms of elevated mortality are found in the northen plains and southern Texas. Similarly, areas of important decreased mortality include the San Francisco Bay area and the urbanized Northeast.

## Discussion

The problems and corrections associated with SHELDUS data raise questions about our decision to use this data source over the CDC's *Compressed Mortality File *as was done by Thacker et al. [4]. No dataset is perfect, and the *Compressed Mortality File*, also has its share of problems. First, unlike *Storm Data *(upon which SHELDUS is based), the *Compressed Mortality File *is not solely focused on natural hazard events. Although both SHELDUS and the *Compressed Mortality File *likely suffer from undercounting hazard related deaths [4,39], it is known that the only reason any of the deaths appear in *Storm Data *(and SHELDUS) is because of some natural event. In the CDC's *Compressed Mortality File*, deaths are interpreted from classifying the underlying cause listed on death certificates [4], whereas SHELDUS mortality is derived from *Storm Data*. NCDC, the parent source for *Storm Data*, uses death estimates for hazard events that may or may not be verified [51]. The accuracy of these estimates is unknown, but some level of undercounting is almost certain. However, *Storm Data *remains the premier data source for weather hazard related losses and deaths [52].

Second, the coding system used by the CDC underwent a major revision after 1998, providing additional and more specific categories for deaths attributed to natural hazards. When undertaking a longitudinal study such as this, any new classification scheme creates analytical problems by introducing a change in the specificity of the data structure. This is shown in the work by Thacker et al. [4] as they were able to use only six types of natural hazards in their analysis because the 1979 – 1998 data were not as detailed as those from 1999 – 2004. Thacker et al. [4] also mentioned that apparent increases in the number of deaths for some natural hazard events might be a statistical artifact of this classification change rather than an actual increase in hazard deaths. Unlike the CDC data disparity, the inconsistencies in SHELDUS before and after 1995 were addressed in later versions of the database.

**Figure 5:**
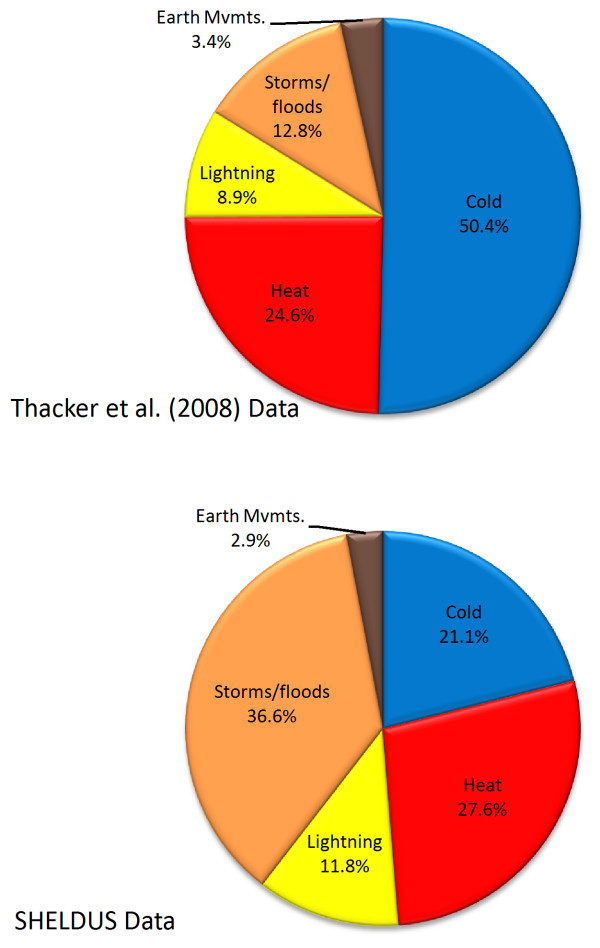
Comparison of deaths by hazard type using Thacker et al. 2008 (A) and SHELDUS (B).

To create a more meaningful comparison between the two datasets, SHELDUS data were modified temporally and categorically to mimic the Thacker et al. [4] study. We extracted a subset of the SHELDUS data for 1979 – 2004, and grouped hazards according to the categories used by Thacker et al. [4] (Table 4). A discrepancy between databases lies in pairing "cold" and "storms/floods" from Thacker et al. [4] with matching categories from SHELDUS. For Thacker et al.'s [4] "cold," the SHELDUS category "winter weather" was used, which includes cold related *and *other winter weather deaths such as those from blizzards and winter storms. These winter storms would technically be categorized under "storms/floods" in Thacker et al. [4]. In SHELDUS, however, cold and winter storm deaths all fall under "winter weather." The proportions of deaths by hazard type seem quite different, with over half of Thacker et al.'s [4] deaths attributed to cold, and the majority of SHELDUS deaths in storms/floods (Figure 5). The proportional differences between Thacker et al. [4] and our data by individual causal agent are statistically significant (p < .01) with the exception of earth movements which is statistically significant at p < 0.05.

**Table 4:** Matching hazard categories between SHELDUS and Thacker et al. (2008)

**Thacker Category**	**SHELDUS Category**
Cold	Winter Weather
Heat	Heat
Lightning	Lighting
Storms/floods	Coastal
	Flooding
	Hurricane/Tropical Storm
	Severe Storm/Thunderstorm
	Tornado
Earth Movements	Avalanche
	Earthquake
	Landslide
	Volcano

Hazard mortality data are fraught with inconsistencies across databases. Differences manifest themselves from the subjective nature of attributing any death to a hazard event. Because of the lack of a standardized death classification scheme [53], hazard deaths are not counted in the same way for any two databases. In fact, even within a national database (i.e. SHELDUS, *Compressed Mortality File*), hazard death attribution likely varies geographically. Therefore, we are cautious that the analyses and conclusions drawn from hazard mortality data are based on estimates of deaths from natural events.

## Conclusion

There is considerable debate about which natural hazard is the most "deadly". According to our results, the answer is heat. But this finding could change depending on the data source, or how hazards within a data source are grouped, as we've shown here. Even if researchers could definitively assert the 'deadliest hazard,' a better issue to pose is *where *residents are more susceptible to fatalities from natural hazards within the United States.

The spatial patterns revealed in the results are not surprising – greater risk of death along the hurricane coasts, in rural areas, and in the South – all areas prone to natural hazards as well as significant population growth and expansion throughout the study period. However, the interpretation of these patterns reveals the problems associated with rare causes of death. Using this analysis as a blueprint for hazard mortality 'hot spots' supports justification for a more in-depth study of hazard- induced deaths in specific regions or communities. It is at this local scale where defining the deadliest hazard becomes important and emergency management officials can take action to try to reduce the number of future deaths.

There are limitations to this study (and others that study mortality from a rare cause of death) that are worth noting. First, we were able to visualize the spatial variation of hazard related deaths for our study period for the entire U.S., but in any given year or in any given county, very few if any deaths may occur. This rarity of occurrence prevented our analysis from providing detailed information on hazard-specific mortality rates or SMRs. Calculation of such measures would be highly unstable due to the minimal number of deaths in many counties [See [4]]. Second, there are limitations in the original data sources as noted earlier.

This paper provides the foundation of a solid understanding of the geography of hazard related mortality over time. Future research can use this information to study specific areas of elevated hazard mortality, and study its correlates in different areas. Ultimately, greater local knowledge about which types of hazards are deadliest in different geographic regions is useful information for strategies aimed at reducing the risk of death from natural hazards.

The over-arching contribution of this work is not to compare and contrast datasets, or even create the "best" possible map of hazard deaths. Rather, this work enables research and emergency management practitioners to examine hazard deaths through a geographic lens. Using this as a tool to identify areas with higher than average hazard deaths can justify allocation of resources to these areas with the goal of reducing hazard deaths. One logical avenue in achieving this goal is to assess the efficacy of information dissemination from emergency managers to the public. An important question is whether people in areas of high mortality know what to do (or what *not *to do) when a hazard event occurs. Improved understanding of how to react in a hazard event will contribute to reduced deaths from hazard events in high-mortality areas.

## Competing interests

The authors declare that they have no competing interests.

## Authors' contributions

SLC conceived of the study, while KAB conducted the research. SLC and KAB wrote the manuscript and the revision. Both authors approved the final manuscript.
